# Antioxidant Therapies and Oxidative Stress in Friedreich’s Ataxia: The Right Path or Just a Diversion?

**DOI:** 10.3390/antiox9080664

**Published:** 2020-07-24

**Authors:** Laura R. Rodríguez, Tamara Lapeña, Pablo Calap-Quintana, María Dolores Moltó, Pilar Gonzalez-Cabo, Juan Antonio Navarro Langa

**Affiliations:** 1 Department of Physiology, Faculty of Medicine and Dentistry, Universitat de València-INCLIVA, 46010 Valencia, Spain; Laura.Robles@uv.es (L.R.R.); lula4@alumni.uv.es (T.L.); pablo.calap@uv.es (P.C.-Q.); 2 Associated Unit for Rare Diseases INCLIVA-CIPF, 46010 Valencia, Spain; 3 Centro de Investigación Biomédica en Red de Enfermedades Raras (CIBERER), 46010 Valencia, Spain; 4 Department of Genetics, Universitat de València-INCLIVA, 46100 Valencia, Spain; Dolores.Molto@uv.es; 5 Centro de Investigación Biomédica en Red de Salud Mental (CIBERSAM), 46100 Valencia, Spain; 6 Institute of Zoology, Universitaetsstrasse 31, University of Regensburg, 93040 Regensburg, Germany

**Keywords:** Friedreich’s ataxia, clinical trials, oxidative stress, antioxidant therapies, reactive oxygen species, scavengers, antioxidant response, mitochondrial metabolism, ferroptosis

## Abstract

Friedreich’s ataxia is the commonest autosomal recessive ataxia among population of European descent. Despite the huge advances performed in the last decades, a cure still remains elusive. One of the most studied hallmarks of the disease is the increased production of oxidative stress markers in patients and models. This feature has been the motivation to develop treatments that aim to counteract such boost of free radicals and to enhance the production of antioxidant defenses. In this work, we present and critically review those “antioxidant” drugs that went beyond the disease’s models and were approved for its application in clinical trials. The evaluation of these trials highlights some crucial aspects of the FRDA research. On the one hand, the analysis contributes to elucidate whether oxidative stress plays a central role or whether it is only an epiphenomenon. On the other hand, it comments on some limitations in the current trials that complicate the analysis and interpretation of their outcome. We also include some suggestions that will be interesting to implement in future studies and clinical trials.

## 1. Introduction

Friedreich’s ataxia (FRDA, OMIM no. 229300) is an autosomal recessive neurological disorder. It is the most common inherited ataxia in Europe, with a prevalence of around 1 in 30,000, although it shows large regional differences [1]. The disease is mostly affecting individuals of Indo-European and Afro-Asiatic origin, whereas the incidence on Amerindians, sub-Saharan, Chinese, Japanese, and Southeast Asiatic populations seems to be residual [2]. Most of the cases (96%) of FRDA are associated with a homozygous GAA triplet repeat expansion in intron 1 of the *FXN* gene, located on 9q21.11 [3]. The remainder are compound heterozygous, with an expansion in one allele and a point mutation, insertion or deletion in the other [4]. Consequently, FRDA patients show decreased levels of frataxin, the protein encoded by the *FXN* gene [5]. Longer expansions are associated with and earlier onset and more severe phenotypes [6,7,8]. Frataxin is a small protein highly conserved throughout evolution [9]. Human frataxin is expressed in the cytoplasm as a precursor of 210 amino acids (23 kDa) and then transported into the mitochondria due to a mitochondrial import signal in the C terminus [10]. Maturation of frataxin involves a two-step process by the mitochondrial processing peptidase leading to the formation of the form of 130 amino acids (14 kDa) that is the most abundant in controls and patients [10]. Mature frataxin lies within the mitochondrial matrix in association with the inner mitochondrial membrane. Frataxin mRNA is mainly expressed in tissues with high metabolic rate such as liver, kidney, neurons and heart [5]. 

Although the function of frataxin remains unclear, early studies observed a deficient activity of the iron–sulphur clusters (ISC) containing enzymes of the mitochondrial respiratory chain and Krebs cycle in heart homogenates, mitochondria from skeletal muscles, fibroblasts and lymphoblasts derived from patients [11,12]. In this line, several reports have detailed the function of frataxin as stimulator of ISC biogenesis by facilitating the persulfide transfer [13,14,15]. ISC are prosthetic cofactors, responsible for the activity of different enzymes which are involved in energy metabolism, iron metabolism, purine synthesis and DNA repair. Although, this role of frataxin is currently widely accepted among the FRDA community, some experimental evidences have questioned the real impact of frataxin’s function in ISC biosynthesis on the neurobiology of the disease. Indeed, molecular and physiological defects have been reported in FRDA models even when deficits in mitochondrial ISC are still undetectable or absent [16,17,18]. Similarly, recovery of ISC formation without restoring frataxin levels has been recently described [19]. Moreover, frataxin has been suggested to participate in other crucial cellular processes such as controlling cellular iron homeostasis as an iron-storage protein [20], interacting with the electron transport chain to regulate oxidative phosphorylation (OXPHOS) [21,22], delivering ferrous iron to ferrochelatase for heme biosynthesis [23] and reactivating aconitase [24]. More recently, a role for frataxin in calcium homeostasis has been proposed as a key element for the pathobiology of the disease [25,26,27,28]. Therefore, the biological function of frataxin still remains largely obscure.

## 2. The Clinical Spectrum of FRDA

In 1863, Nikolaus Friedreich described a disease characterized by a “degenerative atrophy of the posterior columns of the spinal cord”. Clinically, Friedreich recognized the main features of the disease: Ataxia, sensory neuropathy, scoliosis, foot deformity, and cardiomyopathy [29]. In 1996, with the discovery of the underlying genetic cause of the disease, the clinical spectrum was expanded [30]. 

The typical age of symptom onset in FRDA is between 10 and 15 years. Initial symptoms are gait instability and clumsiness, but patients also develop trunk and limb ataxia, which difficult daily activities. Confinement in a wheelchair is required after approximately 11–15 years from disease onset [7,31]. The progression of the disease is different between individuals. Faster progression is associated with earlier symptom onset. Late-onset FRDA (first symptoms after 25 years) and very late-onset FRDA (first symptoms after 40 years) are atypical phenotypes of the disease with a slower progression [32]. Average age at death was reported to be 36.5 years (range 12–87), being cardiac dysfunction the most frequent cause of death (59%) [33]. 

The severity of the disease has been measured by means of different clinical rating scales. The International Cooperative Ataxia Rating Scale (ICARS; [34]) was initially used but in 2005 a report evaluated the true ability of this scale to quantify the severity of FRDA. This report did recommend to use only the total ICARS score and to abandon the subscales [35]. Because of this, two other scales, the Friedreich Ataxia Rating Scale (FARS; [36]) and the Scale for the Assessment and Rating of Ataxia (SARA; [37]) were stablished. More recently a modified version of FARS (mFARS), in which some items have been suppressed and the focus falls on functional abilities of the patients, has been developed [38] and endorsed by a recent study with more than thousand participants [39]. 

### 2.1. Neurological Features 

The wide neurological pathology of FRDA is due to alterations in both the central and peripheral nervous system. Ataxia, the most common symptom in FRDA, occurs because of spinocerebellar degeneration, peripheral sensory neuropathy, and cerebellar and vestibular pathology [40]. The dorsal root ganglia (DRG) are particularly affected in FRDA, showing a smaller size of its neurons and a proliferation of satellite cells. In dorsal roots, there is an atrophy of large myelinated fibers, whereas many thin myelinated axons remained [41]. Severe changes are present in sensory peripheral nerves, producing sensorial neuropathy and posterior column loss with severely reduced or completely absent sensory action potentials [42,43]. It is still unclear whether this peripheral neuropathy is a primary effect of the disease or the result of the lesion in DRG [44]. Consequently, the proprioception is impaired in the patients. The perception of the position, vibration, temperature, pain, and light touch are reduced or lost. Reflexes are absent, particularly in the lower limbs, reflecting the underlying peripheral neuropathy. The progressive degeneration of pyramidal tracts leads to muscular weakness and hypotonia, which suggest that motor nerves are also involved in FRDA [45]. Patients usually show Babinski sign (extensor plant response) and, a lower percentage, also exhibit spasticity [41], that can in turn cause pain, contractures and spasms. The major lesion of the central nervous system is the atrophy of the dentate nucleus in the cerebellum, which occurs as the diseases progresses. Large nerve cells disappear and GABAergic and glutamatergic motor control are lost. Atrophy of the dorsal nuclei of Clarke blocks transmission of spinocerebellar input to the cerebellar hemispheres [46]. Significant grey and white matter in the deep cerebellar nuclei and brainstem are also reduced, according to magnetic resonance imaging and morphometry studies [47,48]. Dysarthria is another primary symptom in FRDA, which is directly related to cerebellar lesions. In advanced stages of the disease, speech becomes unintelligible [49] and FRDA patients start to present a poor capacity in concept formation and visuospatial reasoning [50]. 

Patients can also present hearing difficulties, due to impaired neural conduction in the central auditory pathways [51]. Visual system is involved in FRDA too, with optic nerve involvement, abnormal visual evoked potentials (P-VEPs) and reduced retinal nerve fiber layer thickness [52]. Fixation instability with square-wave jerks (SWJs) is the main oculomotor feature and less frequently nystagmus and saccadic intrusions. Dysphagia is problematic in advanced stages, requiring gastrostomy feeding in some cases. Aspiration pneumonia, related to dysphagia, is an important cause of death [53]. 

### 2.2. Non-neurological Features 

Although FRDA is described as a neurological disorder, it should be mentioned that cardiomyopathy is the main cause of death in FRDA and it is present in a vast majority of the patients. The analysis of 205 patients with different cardiac imaging approaches revealed that neurological scales are unable to predict the severity of cardiac conditions and that age of onset is strongly related to the level of cardiac dysfunction [54]. This aspect has been confirmed in other independent studies. For example, Tsou and coworkers also found that when cardiac dysfunction is the cause of death, those patients displayed hypertrophic traits in earlier stages of the disease course [36]. A main feature of cardiomyopathy in FRDA is chronic myocarditis. Patients show inflammatory cells in the endomysium, attachment of monocytes to cardiomyocytes, and necrosis of heart fibers [55]. Cardiac wall abnormalities, like left ventricular hypertrophy, are also present in most patients, although the ejection fraction is usually preserved [56]. The electrocardiogram is abnormal in almost all cases, with T-wave inversion or flattening in lateral or inferior leads, and ST-segment alterations [57]. Supraventricular tachyarrhythmias can appear in advanced stages of the disease. Among them, atrial fibrillation is the most common and its presence has negative prognostic implications [58]. Furthermore, patients with FRDA have an increased risk of developing diabetes mellitus and glucose intolerance. Diabetes usually appears many years after the first symptoms of FRDA. The mechanism underlying diabetes may be due to a combination of both insulin resistance and insulin deficiency, secondary to pancreatic β-cell apoptosis [59,60]. Moreover, patients show abdominal fatness which correlates with β-cell dysfunctions. Lipogenesis is suggested to be increased in FRDA contributing to insulin resistance [61] that along with the reduced physical activity of the patients can promote metabolic syndrome [62]. Scoliosis is another typical feature for FRDA. Foot abnormalities are also common, specially *pes cavus* and *talipes equinovarus*, which worsen stability and mobility [63].

## 3. Oxidative Stress Markers in FRDA Patients and Models

Oxidative stress was linked to FRDA since the initial molecular studies. The identification of iron deposits in the cardiomyocytes from patients [12,64] and the well-known redox biology of iron via the Fenton reaction [65] were the first elements suggesting such an intimate relation. In this reaction, reduced Fe^2+^ reacts with hydrogen peroxide (H_2_O_2_) to produce oxidized Fe^3+^ and the hydroxyl radical HO^●^. In addition, other reactive oxygen species (ROS), such as superoxide (O_2_^−^) are produced under physiological conditions due to a leakage during the transport of electrons in the respiration process. Therefore, in the context of FRDA, we encounter a situation in which a defective ISC biosynthesis leads to an altered electron transport chain and abnormal OXPHOS activities. These defects increment electron leakage that along with the concomitant surplus of free iron in the mitochondria further contributes to increase ROS levels [66] even in those tissues that display altered metal regulation but no metal accumulations (reviewed in [67]). In agreement, increased levels of malondialdehyde [68] (a lipid peroxidation product), 8-hydroxy-2′-deoxyguanosine [69] (an indicator of oxidized DNA) and higher glutathione transferase activity [70] have been found in vivo in blood samples from patients. Furthermore, additional evidences indicate that frataxin-deficient cells are also unable to recruit antioxidant defenses to counteract ROS effects, which in turn enhances their sensitivity to oxidative stress [71,72]. Oxidative stress effects have been also associated to several other phenotypes observed in FRDA models such as impaired cytoskeletal dynamics [73], abnormal calcium homeostasis, lipid peroxidation [74], and apoptosis [28].

## 4. Reducing Oxidative Stress as a Therapeutic Avenue to Stop FRDA Progression

**Figure 1:**
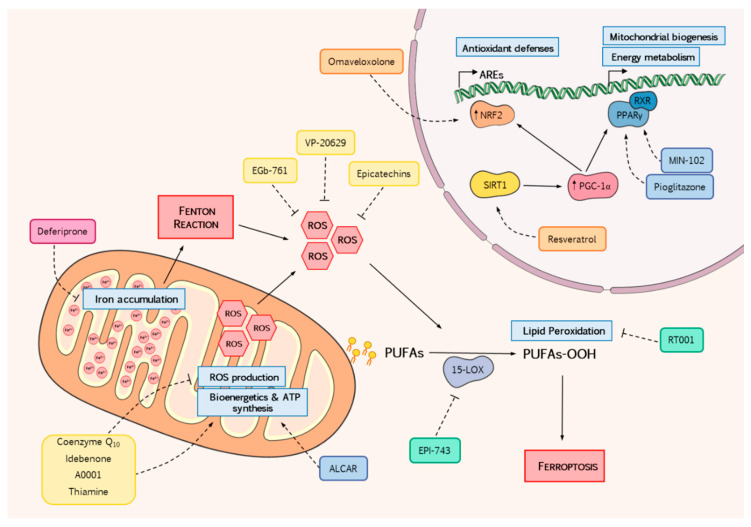
Schematic representation showing the defects associated with frataxin-deficiency that are targeted by the drugs used in the clinical trials summarized in this work. Among others, frataxin deficiency leads to accumulation of mitochondrial iron, a bioenergetic failure of the mitochondria, the impairment of NRF2 to translocate into the nucleus to activate the cellular antioxidant response, an increase in ROS generation that promotes lipid peroxidation initiating the ferroptosis process that culminates with the cell death. Trials analyzed in this review try to determine the effectivity of counteracting this set of events in the patients. Deferiprone reduces mitochondrial iron deposits. Coenzyme Q_10_, Idebenone, A0001, Thiamine and ALCAR exert a double function as antioxidants and promoters of mitochondrial metabolism. EGb-761, VP-20629 and Epicatechins act as ROS scavengers. EPI-743 and RT001 prevent the cellular death by lipid peroxidation (ferroptosis). Omaveloxolone, Resveratrol, Pioglitazone and MIN-102 activate transcription of antioxidant defenses and mitochondrial metabolism via mobilization of transcription factors NRF2 and PPARγ. (NRF2: nuclear factor erythroid 2-related factor 2; PPARγ: Peroxisome proliferator-activated receptor gamma).

The current therapeutic strategies in FRDA can be classified in two main groups, those focused on increasing FXN levels and those focused on alleviating the consequences of frataxin loss. Some of them might belong to both categories (See Table 1, Table S1 and Figure 1 for further information). In this work, we will present an overview of the therapeutic avenues that directly or indirectly aim to mitigate the oxidative stress in frataxin models as well as in patients to improve their conditions and wellbeing. As shown in Table 1, these treatments include antioxidants, iron chelators and nuclear factor erythroid 2-related factor 2 (NRF2) and Peroxisome proliferator-activated receptor gamma coactivator 1-alpha (PGC1-α) modulators. Remarkably, they represent the majority (60%) of all current strategies (when comparing Table 1 and Table S1).

### 4.1. ROS Scavengers

#### 4.1.1. Coenzyme Q_10_ and Idebenone

Coenzyme Q_10_ or ubiquinone is a small lipophilic molecule located in the inner mitochondrial membrane that exerts antioxidant properties as free radical scavenger, preventing the oxidation of cellular structures and maintaining other antioxidant molecules such as vitamins C and E [97,98]. Ubiquinone or its derivates have showed positive effects when applied to fibroblasts from FRDA patients [99].

Almost 20 years ago, an open-label study enrolling 10 FRDA patients was conducted to assess the influence of Coenzyme Q_10_-Vitamin E as antioxidant combined therapy. The outcome parameters included ICARS evaluation and echocardiography. After six months, participants did not show consistent clinical benefits [100]. Authors observed signs of bioenergetic improvement and attributed the short period of study as the reason for the lack of effects, so they continued monitoring the same patients. This long-term longitudinal follow-up study evaluated whether the improvements were sustained for 47 months with the same treatment. There were significant improvements in cardiac and skeletal muscle bioenergetics compared to cross-sectional data from 77 FRDA subjects. Although the posture and gait symptoms continued declining, the ICARS score remained unchanged [101]. A few years later, the same group addressed a double-blind randomized trial in 50 FRDA patients for 2 years. Participants were divided in high-and low-dose groups of coenzyme Q_10_-Vitamin E combined therapy. Interestingly, 49% of the patients who completed the study (21 of 43) showed slower degeneration and clinical improvement leading to an increased ICARS score independently of the doses taken. A closer look into the data highlighted a strong correlation between efficacy of the therapy and decreased baseline Coenzyme Q_10_ and vitamin E levels [102]. 

Idebenone is a structural analogue of coenzyme Q_10_ with lower molecular weight and improved water solubility, which enhances bioavailability. This compound exhibits antioxidant properties as ROS scavenger, lipid peroxidation inhibitor and supports mitochondrial function through the transport of electrons within the mitochondrial respiratory chain, thus increasing ATP production [103]. Interestingly, it has been able to improve longevity, locomotion, and aconitase activity in a fly model of FRDA [104] and delay cardiac phenotypes in a mouse model of the disease [105]. 

The first preliminary clinical study with idebenone performed 20 years ago showed promising improvements of the cardiac outcome in three FRDA patients [106]. Numerous studies followed over the next decade in order to evaluate the effects of idebenone in additional cohorts of FRDA patients. Although, most of them reported cardiac improvements, the results obtained after the analysis of neurological parameters were highly variable (see [75] for further information). Furthermore, four randomized placebo-controlled phase-III clinical trials (NICOSIA, IONIA, MICONOS, and PROTI) did not obtain remarkable evidences on the neurological or cardiac function of idebenone in different groups of FRDA participants [107,108,109,110,111]. Despite these inconclusive results, many FRDA patients are currently using both coenzyme Q_10_ and idebenone as a nutraceutical supplements due to its well tolerance and reduced side effects [112]. 

#### 4.1.2. A0001

A0001 or alpha-tocopherol quinone is a synthetic compound, similar to Coenzyme Q_10_ and idebenone, with enhanced redox potential. It is expected to alleviate mitochondrial oxidative stress, protect against lipid peroxidation and promote electron transfer to the respiratory complex [113]. 

Forty-five participants were enrolled in a double-blind placebo-controlled clinical trial with two doses of A0001 (see Table 1). This compound proved well tolerance and no major adverse effects were detected. Primary clinical outcomes included glucose-handling measures but did not show major differences for any of the groups. Importantly, FARS score improved significantly in a dose-dependent manner, specifically, 4 points above the placebo group [76]. There are no additional studies related to A0001 despite these encouraging results [16].

#### 4.1.3. EGb-761

EGb-761 is a Ginkgo biloba extract that has been widely used in Alzheimer’s disease. Despite its neuroprotective effects described in in vitro and in vivo Alzheimer models, EGb-761 failed to show any substantial benefits in patients, leading to controversy [114]. A phase II, randomized, placebo-controlled double-blind study evaluated the efficacy of EGb-761 in 22 FRDA participants. However, no major differences were found between both groups. Authors claimed the number of individuals was not sufficient to perform the statistical analysis, therefore the study was dismissed [78]. 

#### 4.1.4. VP-20629

Indole-3-propionic acid or VP-20629 (also known as SHP622, OXIGON, or OX1) is present in human plasma and cerebrospinal fluid as a result of tryptophan metabolism by gut microbiota. It has demonstrated a potent hydroxyl radical-scavenging activity, mitigating lipid peroxidation and exerting neuroprotective properties in cellular and animal models of ischemia and Alzheimer’s disease [115,116]. Although VP-20629 was initially developed as a treatment for Alzheimer’s disease due to its ability to inhibit beta-amyloid fibril formation [117], a multicenter randomized, double-blind placebo-controlled phase I trial enrolled 46 FRDA participants to evaluate its safety and tolerability. While all doses administered (see Table 1) were well tolerated and minor adverse events were reported, this compound failed to exert major benefits [77]. 

#### 4.1.5. (+)-Epicatechin

Catechins are polyphenolic flavonoids found in fruits, red wine and cocoa whose mechanisms of action exert either direct or indirect antioxidant effects. These compounds act as ROS scavengers, metal ion chelators and promote the activity of antioxidant enzymes, also inhibiting pro-oxidants ones. In vitro studies performed with catechins revealed a protective role in neurons from oxidative stress and the promotion of mitochondrial function (reviewed in [118]). These results suggested that catechins might have potential benefits in reducing neurodegenerative processes.

An open-label study was conducted to evaluate the safety and efficacy of (+)-Epicatechin in 10 FRDA participants for 24 weeks. Primary outcome measures included changes from baseline in FARS score and changes in ventricular hypertrophy. The results have been recently published and indicate that the drug was well tolerated and was able to improve the mean left ventricle ejection fraction as a readout of cardiac function. However, no improvements of FARS/mFARS scores were observed [79]. 

#### 4.1.6. Thiamine

Thiamine (vitamin B1) is a specific cofactor of many enzymes involved in the energetic metabolism and also plays a non-cofactor role as ROS scavenger. Thiamine deficiency results in severe neurological alterations in the central and peripheral nervous system [119]. Molecular and clinical alterations of thiamine deficiency, such as impaired oxidative metabolism, increased oxidative stress and selective neuronal loss, are similar to those described in FRDA [120]. In addition, significative decreased levels of thiamine were measured in the cerebrospinal fluid of FRDA patients [121,122]. An open-label trial evaluated the influence of thiamine in 34 FRDA patients and reported significant SARA score improvements and recovery of swallowing difficulties, tendon reflexes and interventricular septum thickness. Despite these results, the absence of a placebo group and further studies about the role of thiamine in FRDA led to discard this compound as therapeutic candidate [80]. 

### 4.2. Promotion of Antioxidant Response 

#### 4.2.1. NRF2 Inducers

The NRF2 is a transcription factor responsible for the expression of antioxidant enzymes that share a common promoter sequence called the antioxidant response element (ARE). For this reason, NRF2 is considered a master regulator of the antioxidant response. Under homeostatic/physiological conditions, NRF2 is located in the cytoplasm where it is regulated by its inhibitor Kelch-like ECH-associated protein 1 (KEAP1). KEAP1 works as an adapter for CUL3/RBX1-mediated degradation of NRF2 via proteasome. Under oxidative stress conditions, NRF2 is stabilized and translocates to the nucleus, where activates the transcription of ARE-containing genes, maintaining a correct redox state in the cell (for further information about the regulation of NRF2 see [123,124]).

Elevated levels of KEAP1 have been observed in FRDA models, which in turn increases NRF2 ubiquitination and its subsequent degradation [125]. In addition to reduced levels of functional NRF2, other authors have observed an impairment of its translocation to the nucleus in response to oxidative stress [72,126] with some exceptions [127]. It is likely that abnormalities in the actin filaments participate in the mechanism affecting the subcellular distribution of NRF2 [72,128]. Accordingly, the expression of numerous cytoprotective genes is decreased and the antioxidant response is compromised, enhancing cellular hypersensitivity to oxidative insults. Moreover, NRF2 has been described to regulate mitochondrial biogenesis [129]. In agreement, reduced NRF2 levels also result in imbalanced mitochondrial potential membrane, ATP production and respiration in frataxin-deficient cells [130]. 

##### Omaveloxolone 

Omaveloxolone or RTA-408 is a small molecule that activates NRF2 function [131] and modifies the inhibitory action of KEAP1 over NRF2 (reviewed in [132]). Compared to the finite potential of other antioxidants, this compound is capable of activating antioxidant defenses as well as mitochondrial bioenergetics. Abeti and coworkers reported encouraging benefits of omaveloxolone in different in vitro FRDA models, reverting the pathological phenotype by reducing lipid peroxidation, mitochondrial ROS production and preventing oxidative-stress induced cell death [133]. Omaveloxolone was assessed in a two-part multicenter, double-blind, randomized, placebo-controlled trial designated MOXIe. The first part of the study, revealed well tolerance, mild adverse effects and dose-dependent improvements in the mFARS score [81]. The second part includes a parallel group study to evaluate the safety and efficacy of 150 mg of omaveloxolone [82]. Although results are still pending publication after a peer reviewed process, the pharmaceutical company that sponsors this trial announced that omaveloxolone significantly improved the mFARS score after 48 weeks of treatment. Remarkably, this is the first study in FRDA to achieve its primary endpoint [83]. Now the company will proceed with the necessary steps for marketing approval.

##### Resveratrol

Resveratrol is a polyphenolic phytoalexin found in several plant species that exerts antioxidant, anti-inflammatory, anti-apoptotic and neuroprotective properties. This versatile molecule participates in a wide variety of cellular processes, including the activation of the NRF2, PGC1-α and NAD-dependent deacetylase sirtuin-1 (SIRT1), thus promoting mitochondrial biogenesis and antioxidant defenses [134]. 

Li et al. reported that resveratrol significantly promoted frataxin expression in Hela cells, lymphoblasts from FRDA patients and mouse brain from the YG8R FRDA model [135]. Similar results were observed in a second study that used fibroblasts derived from patients [136]. However, when the authors explored the sensitivity to resveratrol in patient-derived induced pluripotent stem cells differentiated into mesenchymal and neuronal cells, they detected mild or no effect, respectively [136]. Due to these cell-specific responses, authors described resveratrol as a non-appropriate treatment for the disease. In agreement, an open-label clinical trial that evaluated two doses of resveratrol (low and high, see Table 1) in 27 participants for 12 weeks also showed that frataxin levels in peripheral blood mononuclear cells remained unchanged in both groups. Interestingly, the high-dose resveratrol treatment was able to decrease the lipid peroxidation biomarker F_2_-Isoprostane. Also, FARS and ICARS scores showed improvements, especially in neurological features. Unfortunately, frequent gastrointestinal side effects were observed in the group of patients treated with high-dose resveratrol. As a result, the authors highlighted the necessity of enhancing tolerability and bioavailability of the compound [84]. In this line, a double-blind, randomized placebo-controlled crossover clinical trial is currently ongoing to assess the efficacy of a micronized resveratrol. The primary outcome measures will include the mFARS scale [85]. 

#### 4.2.2. Mitochondrial Metabolism

Metabolic perturbations due to reduced frataxin levels have been described in the FRDA literature [60,74,137,138,139,140,141]. Several downstream pathways such as β-oxidation of lipids, peroxisome proliferator-activated receptor gamma (PPARγ) signaling, lipogenesis via upregulation of the sterol-responsive element-binding protein 1 (SREBP1) or inhibition of the mitochondrial NAD^+^-dependent deacetylase sirtuin-3 (SIRT3) [141,142,143] have been proposed as contributors, but molecular mechanisms still need to be fully elucidated. 

##### Pioglitazone and Leriglitazone 

PGC-1α is a transcriptional regulator that plays a main role in mitochondrial biogenesis and energy homeostasis. It is strongly induced at the transcriptional level by factors such as cold exposure, exercise and cytokines produced by stress conditions, glucagon or catecholamines through the PKA, p38 MAPK, cAMP, and calcineurin pathways [144]. PGC-1α is then recruited to modulate the expression of several genes involved in metabolic pathways including nuclear respiratory factors (NRFs) or peroxisome proliferator-activated receptors (PPARs) [145]. PPAR-γ is a hormone nuclear receptor whose activation depends on PGC-1α binding. PPAR-γ activation is related to glucose homeostasis, lipid storage, fatty acid oxidation or differentiation of adipocytes in brown adipose tissue [146].

A microarray analysis of skeletal muscle and liver from frataxin-depleted mice revealed reduced activity of PGC-1α. PGC-1α expression as well as the levels of PPAR-γ were corroborated in a myoblast model, in a second mouse model and cells derived from FRDA patients. Interestingly, downregulation of this pathway further affects frataxin expression due to a feedback loop [61]. PGC-1α down-regulation is associated to impaired mitochondrial biogenesis and fatty acid β-oxidation, which may lead to the accumulation of lipids and, ultimately, insulin resistance [147]. Furthermore, reduced expression of PGC-1α has been also related to lower levels of antioxidant enzymes such as of superoxide dismutase 2 (SOD2) in neurodegeneration models [148] including FRDA [149], thus decreasing the cellular ability to control oxidative stress. Because of this, PPAR-γ agonists, like thiazolidinediones (TZDs), have been proposed as a treatment for FRDA. Remarkably, they have been shown to promote mitochondrial biogenesis, antioxidant defense and neuronal survival, and reduce inflammation in many model systems [150]. Although TZDs have been mainly used as antidiabetic drugs [151], they are currently being tested as a treatment for neurodegenerative diseases such Alzheimer, Parkinson, or Huntington’s disease [150]. 

Pioglitazone is a PPARγ agonist that significantly increased the antioxidant response in fibroblasts from patients and in the knock-in/knock out (KIKO) FRDA mouse model by promoting the expression of SOD2 and PGC-1α [149]. A prospective, randomized, double-blind clinical trial was conducted with the objective of exploring the effects of pioglitazone over neurological defects. The study was completed in 2013, but unfortunately the results are not yet available [86], which might suggest a lack of efficacy of this molecule. Leriglitazone or MIN-102 is a selective metabolite of pioglitazone indicated for diseases of the central nervous system. This compound increases DRG neurons survival, mitochondrial biogenesis, and bioenergetics and reduce oxidative stress and inflammation in the KIKO and YG8sR mice and fibroblasts from FRDA patients [152,153]. The FRAMES clinical study has recently started with 36 FRDA patients. It is a phase II, randomized, double-blind, placebo-controlled trial that will evaluate the effects of MIN-102 on biochemical, imaging, neurophysiological, and clinical markers [87]. Although no results are already available from the clinical trials with this couple of TZDs, it is important to mention that they might exacerbate the cardiovascular problems in FRDA patients as suggested from their ability to reduce cardiac fast fibers or to induce a thrombotic response [154]. 

##### Acetyl-l-Carnitine 

Acetyl-l-carnitine (ALCAR) is an enhanced version of l-carnitine since the inclusion of the acetyl group increases water solubility and bioavailability. It is a mitochondrial cofactor considered as a good candidate for neuroprotective therapies in several clinical trials. It provides essential chemical groups for energy metabolism and has demonstrated anti-inflammatory and antioxidant properties [155]. This family of compounds was firstly assessed more than a decade ago in 16 FRDA patients with the aim to enhance mitochondrial energy imbalance. This was a double-blind, placebo-controlled, crossover trial that tested a combined therapy of l-carnitine and creatine. Although both compounds were well tolerated, there was no positive impact in FARS score and echocardiographic data in the treated group compared to the placebo group [88]. An open-label trial using the improved version ALCAR was conducted few years ago including cardiovascular outcome measurements. However, results are not yet available [89]. 

### 4.3. Counteracting ROS Production

#### 4.3.1. Iron Chelators 

As already stated before, the potential role of frataxin as an activator of mitochondrial ISC biogenesis might underlie the iron accumulation in the mitochondria upon frataxin deficiency. This is considered one of the hallmarks of FRDA and a pivotal pathogenic consequence of frataxin depletion. The loss of iron homeostasis in patient’s samples and FRDA models is not only reflected as the presence of iron deposits [12,156,157,158]. It can be also observed as alterations in the iron content or iron redistribution [67]. Other evidences such as changes in the expression of iron related proteins support an iron dyshomeostasis that would result in its accumulation in the mitochondria along with a concomitant depletion in the cytoplasm [18,159,160,161]. However, lack of alterations in the iron levels are also found in the literature [31,162,163,164,165]. This unexpected and significant variability of data among patients and models questions the true relevance of the accumulation of iron in the development and in the timing of either the cardiac or the neurological manifestations of the disorder. 

Cellular iron can be found in its reduced (Fe^2+^) or in its oxidized (Fe^3+^) form and most of it is stored or bound as a prosthetic group to different proteins. However, a small proportion of less than 5% is found in the redox-active iron labile pool [166]. An increase in the amount of this free redox-active iron in FRDA is especially relevant, since it can generate highly toxic ROS via the Fenton and Haber–Weiss reaction [167]. Among all ROS, hydroxyl radicals are highly reactive, more than superoxide radicals or hydrogen peroxide. Furthermore, such radicals can damage proteins, lipids, and DNA through oxidation. Finally, Fe^3+^ is reduced back to Fe^2+^ by the superoxide radical O_2_**^−^** produced mainly in the mitochondria Despite the toxic outcomes derived from this iron dyshomeostasis, the number of therapeutically approaches aimed to improve it has been scarce being deferiprone the most relevant compound tested.

##### Deferiprone

Deferiprone is a lipid-soluble membrane-permeable iron chelator which can cross the blood-brain barrier and cellular membranes. It has been successfully used to treat iron overload in hemoglobinopathies [168]. Remarkably, it is capable of reaching mitochondrial iron deposits in all tissues including nervous system [169,170]. This chelator also has low affinity for iron and can transfer it to transferrin and to other cellular acceptors [171]. All these properties confer deferiprone promising therapeutic characteristics as a candidate compound for FRDA, since it removes iron surplus without inducing iron depletion [172]. 

An in vitro assay performed with frataxin-depleted cells demonstrated deferiprone reduced energetic defects and slowed oxidative destruction of ISC [173]. In agreement, Soriano et al. reported improvement of longevity and motor abilities in a frataxin-deficient model of *Drosophila melanogaster* due to deferiprone treatment [104]. Several clinical trials have been conducted regarding deferiprone assessment in FRDA, without consistent outcomes. A randomized, double-blind, placebo-controlled trial assessed the safety, tolerability and efficacy of three doses of deferiprone (20, 40, and 60 mg/kg/day). The highest dose was removed because of adverse effects that worsened ataxia in two patients. Interestingly, FARS score was stabilized in the lowest dose and worsened in the intermediate and significant improvements were reported in cardiac hypertrophy with 20 and 40 mg/kg/day [90]. An open-label single-arm study enrolled 20 patients to evaluate a combined therapy of idebenone and deferiprone for six months. No outstanding effects were found in ICARS score, but echocardiographic parameters showed significant differences again. Patients experienced mild adverse effects and two of them presented reversible neutropenia restored after drug withdrawal [91]. A triple-combined therapy was implemented in an open-label study evaluating the efficacy of deferiprone, idebenone, and rivoflavin. There were inconclusive results concerning neurological and cardiological measurements in addition to deferiprone-related adverse effects [92]. The absence of neurological improvement highlights the necessity of further investigations to reevaluate deferiprone as a therapeutic molecule for FRDA. It is possible that the ability of deferiprone to target aconitase activity in controls and FRDA cells [174] may contribute to the outcome of the clinical trials using this compound.

#### 4.3.2. Lipid Metabolism

A recent study in isolated platelets from FRDA patients has reported an increased lipid metabolism which strongly supports a metabolic switch in the patients [138]. Such a switch might precede other deregulations in lipid uptake, synthesis, storage, and utilization. Indeed, alterations in lipid metabolism, mostly linked to accumulations, have also been observed in mouse [175], cultured cells [141], *D. melanogaster* [74], and Caenorhabditis elegans [176]. On the other hand, studies from patient autopsies or plasma samples showed contradictory results regarding changes in total cholesterol, triacylglycerides, and fatty acids profiles [177,178]. Alterations of lipid metabolism might represent a critical issue in FRDA because fatty acids are extremely sensitive to oxidative modification, resulting in the formation of lipid peroxides, which are highly cytotoxic and promote damage to other lipids, proteins and DNA [179]. Moreover, the oxidation of polyunsaturated fatty acids (PUFAs) leads to the generation of lipid peroxide breakdown products that can act as signals in this kind of cell death [180]. Indeed, frataxin-deficient cells and models also display increased levels of markers of lipid peroxidation [68,74,106,175,181]. In agreement, scavengers of lipid peroxides have been shown to improve frataxin-deficient conditions [74,181]. These evidences suggest lipid metabolism as a primary target of frataxin deficiency and place lipids as central elements in the pathology of FRDA. 

Lipid peroxidation and the mitochondrial iron accumulation converge as hallmarks of a newly described form of regulated cell death named ferroptosis [182]. Ferroptosis is associated with a reduction in the detoxification of lipid peroxides by the glutathione peroxidase 4 (GPX4), being the peroxidation of PUFAs a main factor in this process of cell death [183]. Depletion of GSH or imbalance in the oxidized/reduced glutathione (GSSG/GSH) pools have been observed in some FRDA models [127,184,185,186,187] and patients [188]. Importantly, GPX4 requires GSH for its lipid repair function, reducing GSH to GSSG to reduce lipid hydroperoxides [189]. In agreement, depletion of GSH and loss of GPX4 activity results in accumulation of lipid peroxides and ferroptosis [190]. Furthermore, GSH can bind Fe^2+^ in the labile iron pool to prevent its oxidation and the production of the hydroxyl radical [191] and the subsequent lipid peroxides through the production of ROS in the Fenton reaction [183]. Ferroptosis is strongly linked with several types of cancer but also seems to be a pivotal element in some neurodegenerative disorders [192]. Different in vitro and in vivo FRDA models displayed increased sensibility to this form of cell death and ferroptosis inhibitors such as SRS11-92 and Fer-1 successfully protected FRDA cells against toxic iron concentrations or oxidative insults. This data suggests that such inhibitors might also induce clinical benefits [193]. Remarkably, the known roles of frataxin in iron metabolism and mitochondrial activity suggest that frataxin is instrumental in the regulation of ferroptosis [194]. 

##### EPI-743

Iron can promote ferroptosis as part of the catalytic subunit of iron-dependent lipoxygenases that catalyze the oxidation of fatty acids [195]. In this sense, different iron chelators such as deferoxamine or the knockdown of the iron importer transferrin receptor 1 are able to counteract the induction of ferroptosis by erastin or RSL3 [182,196,197]. EPI-743 or α-tocotrienol quinone is a novel redox therapy that inhibits 15-lipoxigenase activity, a key regulator of inflammation, oxidative stress and ferroptosis [198]. In comparison, EPI-743 exerts from 1000 to 10,000-fold antioxidant potential in vitro over coenzyme Q_10_, idebenone or resveratrol [199].

An open-label study reported the impact of EPI-743 in patients suffering from different inherited mitochondrial diseases, including FRDA. The only FRDA participant, a 17 years-old female, showed improvements in the brain redox state, speech, sight and motor skills throughout the treatment [200]. Following this single result, a double-blind, multicenter trial assessed two doses of EPI-743 in FRDA participants (see Table 1). The study consisted of a 6-month placebo-controlled phase followed by an 18-month open-label phase. As visual loss is a frequent clinical symptom in FRDA patients, retinal nerve fiber layer thickness was included in the primary outcome measurements. The compound demonstrated well tolerance and safety in most patients, without several adverse effects reported. While there were no clinical improvements in sight measurements or echocardiography at 6 months, significant differences in the FARS score were found in the high-dose group compared to the placebo. Thus, patients received high dose of EPI-743 for the second phase of the study. The progression of the disease decelerated when compared with the natural history of the disease at 24 moths, thus indicating significant improvements in neurological function [93]. The pharmaceutical company in charge of this compound includes EPI-743 (now PCT-743) in its pipeline for this year. Registration for a trial in FRDA seems to be still planned for 2020 [94]. 

##### Deuterated Fatty Acids: RT001 

PUFAs are essential fatty acids required for the structure of cell membranes. Several studies have demonstrated that they display anti-inflammatory properties and beneficial effects reducing mitochondrial dysfunction, ROS production and endoplasmic reticulum (ER) stress [201]. However, PUFAs contain carbon-hydrogen bonds prone to be cleaved by ROS, creating a type of ROS that reacts with more PUFAs resulting in a chain reaction that damages cell membranes [179]. 

Cotticelli et al. described promising effects of linoleic and deuterated α-linolenic acids over oxidative-stress challenged FRDA cell models. The replacement of hydrogen (^1^H) with the isotope deuterium (^2^H) strengthens the structure, reducing toxicity and increasing the resistance to lipid peroxidation [202]. Remarkably, deuterated PUFAs (dPUFAs) prevented cell death and lipid peroxidation in fibroblasts from the KIKO and YG8R mouse models [181], paving the way for a clinical test. RT001 (Retrotope) is a deuterated homologue of linoleic acid assessed in a double-blind, placebo controlled clinical study which enrolled 18 FRDA participants for 28 days. This compound proved to be safe and well tolerated (see Table 1). Cardiopulmonary exercise testing showed improvements in the RT001 group compared to placebo one, as well as modest tendencies of neurological recovery [95]. These encouraging results led to further investigations. A randomized, double-blind, placebo-controlled phase III trial focused on the assessment of RT001 is currently recruiting FRDA patients. Outcome measures will include cardiopulmonary exercise testing and mFARS score [96].

## 5. Discussion and Conclusions 

In this review, we have aimed to provide an overall and comprehensive view of the long-lived strategy of combating oxidative stress as an approach to cure or at least slow down the progression of FRDA. We reviewed 35 clinical trials that tested 15 drugs with different characteristics, including antioxidant properties. Unfortunately, the results in many cases have not been published, and thus it is difficult to assess whether the negative outcome of the trial was more related to the experimental design or to the intrinsic properties of the chemical compounds. Remarkably, five compounds have reached phase III in clinical trials. This represents a 14%, which is in the expected success rate when compared to a recent study from Massachusetts Institute of Technology that analyzed 21,000 compounds in different disease groups from 2005 until 2015 [203]. 

Clinical trials in FRDA have to deal with the handicap that FRDA is a rare disease. This hampers the possibility to recruit a number of patients large enough to distribute them in homogeneous groups which in turn has a tremendous impact in the statistical analysis of the results. Another limitation is, in our opinion, the lack of a standardized methodology of data collection in all these clinical trials that compromises the correct compilation of natural histories of patients and their comparison. In addition, the influence of epigenetic and environmental factors in the progression of the pathology further impact on the capability to define the natural histories of patients. The development and consensus of new scales such as SARA [204] and mFARS [39], together with the generation of patient records containing comprehensive clinic and epidemiologic information is essential but results are still limited and dispersed. All these aspects hinder the proper classification of patients for the design and analysis of clinical tests. Overcoming these limitations seems pivotal to reveal whether the treatments display positive impact in FRDA patients with specific features, such as the stage of disease progression, a given length of the GAA repeat expansion, age of onset, and levels of specific metabolites (biomarkers). This approach will facilitate the way to a personalized pharmacological treatment. The need of such a customized process is clearly emphasized by one of the trials performed with the combination of coenzyme Q_10_-Vitamin E. In this trial, the treatment only had a positive influence in patients displaying low endogenous levels of both molecules in blood samples [102]. Another difficulty is driven by the fact that FRDA is a disorder with a relatively slow but relentlessly progression. This might indicate that the molecular consequences of frataxin-deficiency also accumulate in the cell in a very gradual manner and therefore, the reversal process that might be triggered by the treatments follows the same trend. This will prevent the detection of clinical benefits during the short period of clinical trials. 

Identification of easy-to-monitor surrogate end-point measures and disease biomarkers that accurately parallel the natural history of the disease is the cornerstone to design solid clinical trials in which patients will be grouped in a more precise manner. In this sense, some progress is being made with surrogate tissues such as lymphoblasts to quantify expression levels of given mRNA or miRNA as potential markers [205,206,207] and platelets to monitor accurate frataxin levels or glucose/lipid metabolic disturbances in controls and patients [138,208]. However, these studies are based on cell types that might be not relevant to analyze neuronal features that are monitored in the clinical scales. In order to bypass this problem, Gino Cortopassi’s team have compared the transcriptomic signatures of lymphoblasts and fibroblasts from patients with RNA-seq data from murine frataxin-deficient DRG neurons [209]. This approach allowed the identification in all 3 cell types of common alterations, that might become future biomarkers, in genes involved in mitochondrial stress, apoptosis, oxidative stress, and selenium metabolism. Paula Giunti’s group opted for directly analyzing the presence of nervous system derived proteins in body fluids [210]. They found increased levels of plasma neurofilament-light chain and glial fibrillary acidic protein suggesting neuronal degeneration and glial activation. All these findings might be of high relevance for a multisystemic disease such as FRDA. We think that clinical trials should also embrace the same comprehensive view of the disease that the basic research is trying to achieve. Imaging systems might be a good and complementary approach to evaluate the progression of the disease and the response to therapies in sensitive FRDA tissues such as heart and central nervous system (reviewed in [211]). Although more expensive and less available to clinicians than serum biomarkers, imaging approaches will allow to follow the evolution of structural changes. These approaches comprise, among other methodologies, diffusion tractography of white matter, fiber tractography by diffusion tensor imaging, epidermal nerve fiber density via skin biopsies, and corticokinematic coherence using magnetoencephalography, myocardial perfusion reserve with cardiac magnetic resonance imaging, and heart bioenergetic via cardiac magnetic resonance spectroscopy, as well as changes in metabolites (*N*-acetylaspartate (tNAA), myo-inositol and iron). 

Nevertheless, it is striking that many treatments aimed to counteract the burden of oxidative stress, one of the best-described downstream consequences of frataxin deficiency, are generating such poor results. Although it is well documented that loss of frataxin leads to an increased in oxidative stress markers, several studies using the same model systems failed to detect the boost in ROS (reviewed in [212]). Remarkably, another phenotype conserved in yeast, flies and mammalian cellular models is the hypersensitivity of frataxin-deficient cells to oxidative stress [213,214,215], likely due to their inability to handle oxidative insults as already described in this review. It is possible that this alternative view of the oxidative stress problem in FRDA points towards a better route to discover promising therapies. Indeed, most of the trials that have been completed without a clear positive impact on patient wellbeing, involved drugs that aim to reduce ROS production. Importantly, the trials that are based on drugs focused on improving the ability of frataxin-deficient cells to deal with oxidative stress are still ongoing (Table 1) and results will be very informative to evaluate this hypothesis and to design new treatments. Furthermore, there are still some interesting options to test in future clinical trials. For example, dimethyl fumarate is the most successful NRF2 activator up to date and it might be of interest according to results in FRDA models [216].

## 6. Future Directions and Prospects 

The discouraging results obtained in the clinical trials using drugs that exert direct or indirect effects on the cellular redox status might indicate that there are other pathological elements at play that are not targeted by these therapies. Additional pathways have been suggested to be involved in the molecular pathology of FRDA. ER stress, calcium-dependent mitochondrial dysfunction, metal metabolism beyond Fenton reaction and hypoxic response are cellular processes strongly related to the iron metabolism and the energetic failure of the mitochondria. The activation of ER stress response in FRDA is consistent with different hypotheses, such as aggregation of misfolded Fe-S proteins, redox alterations, and abnormal calcium metabolism [217]. Administration of compounds that target the ER stress has been successful in fly models of the disease [218]. Interestingly, calcium deposits have also been observed in cardiac cells of FRDA patients [219]. Defects in calcium homeostasis are reported in several in vitro studies in multiple cellular FRDA models [25,26,27,28,220,221]. Remarkably, modulation of calcium signaling by means of calcium chelating agents is able to ameliorate FRDA defects [26,220,222]. Cytosolic iron starvation is present in mammalian and in invertebrate FRDA models [18,164,223]. Such a scarcity activates in turn an evolutionary-conserved response mediated by hypoxia inducible factors [164,217,223,224]. In this sense, a recent work shows that in 4 different models (mouse, human cells, *C. elegans* and yeast) low oxygen conditions restore ISC biosynthesis, iron availability, the activation of NRF2 and the ER stress response and attenuates the progression of the disease [19]. It would be of high interest to monitor if exposure of FRDA patients to controlled periods on normobaric hypoxia shows any improvement. Besides iron, it has been suggested that metal dysregulation in FRDA affects other metals [225]. Importantly, zinc and copper redistribution has been reported in the dentate nucleus of the cerebellum in FRDA patients [226]. The role of these metals in the FRDA pathology is completely unknown and therefore, the impact of a therapy focused on controlling cellular levels of these metals has not been addressed with the exception of a work using the fruit fly [225]. Among these metals, zinc might be of special relevance due to its ability to impair ISC assembly [15]. 

Finally, we think it is pivotal to promote the progression on treatments based on increasing frataxin levels (Table S1) and on gene replacement [227,228] and stem cell-based therapies (reviewed in [229]), since some improvements have been already observed in human patients with this last approach [230]. Besides that, it should also be explored the effect of cocktails containing compatible drugs. Many trials are based on administration of single drugs as shown in this review. Therefore, analysis of synergic effects arising from the combination of several compounds that might revert simultaneously different downstream effects of frataxin deficiency (Figure 1) is an interesting avenue that it is worth to explore in both, disease models and patients. 

## Figures and Tables

**Table 1:** Summary of clinical trials carried out and ongoing in FRDA patients with compounds that target oxidative stress at different levels. Trials have been grouped according to the mechanism of action of the drug tested. To facilitate comparison, table also includes the range of doses used, the corresponding phase of each trial, its result when available and the present or future prospect of the treatment. (CoQ_10_: Coenzyme Q_10_). The clinicaltrial.gov reference numbers for trials that are currently ongoing have been provided.

Compound	Mechanisms of Action	Doses	Clinical Trials	Study Outcomes	Current State
**Direct Reactive Oxygen Species (ROS) Scavengers**					
CoQ_10_	Mitochondrial cofactor Radical scavenger Maintains antioxidant molecules	30–600 mg/day	Several clinical trials have been conducted. Reviewed by Parkinson et al. [75]	Combined therapy of CoQ_10_-vitamin E improved neurological function and bioenergetics in patients with deficiency in CoQ_10_ or vitamin E	No further investigation
Idebenone	Structural analogue of CoQ_10_ with enhanced bioavailability	2.5–75 mg/kg/day 360–2250 mg/day		Variable results in neurological and cardiac function. Inconclusive benefits	No further investigation
A0001	Enhanced version of CoQ_10_ High antioxidant potential Alleviates mitochondrial ROS Protects from lipid peroxidation Electron donor to the respiratory complex	1–1.5 g/day	Phase II, double-blind, placebo-controlled 4-week study with two doses [76]	No differences in primary outcomes (glucose-handling) but significant improvements in Friedreich Ataxia Rating Scale (FARS) score	No further investigation
VP-20629	ROS scavenger Alleviates lipid peroxidation Neuroprotective	150–1200 mg/day	Phase I, randomized, double-blind, placebo controlled with single or multiple dose for 7 days [77]	Safe and well tolerated. No major benefits reported	No further investigation
EGb-761	Neuroprotective Antioxidant	240 mg/day	Phase II, randomized, placebo-controlled, 3-months, double blind study [78]	No significant differences were found. Insufficient number of individuals	No further investigation
Epicatechins	ROS scavenger Metal ion chelator Promotes antioxidant activity Inhibits Por-oxidant activity	75–150 mg/day	Phase II, open-label, prospective, 24-weeks, single center study [79]	Improvement in cardiac structure and function. No significant changes in neurological outcomes	No further investigation
Thiamine	Specific cofactor in energetic metabolism ROS Scavenger	200 mg/week	Open-label trial for 2 years [80]	Improvements in Scale for the Assessment and Rating of Ataxia (SARA) score, cardiological outcomes and recovery of motor skills	No further investigation
**Nrf2 Inducers**					
Omaveloxolone (RTA-408)	Increases antioxidant defenses Reduces Lipid peroxidation and mitochondrial ROS production Ameliorates mitochondrial energy imbalance Prevents from oxidative stress	2.5–300 mg/day	Phase II/III, multicenter, randomized, placebo-controlled, double-blind, 12-weeks, dose-escalation trial (Part 1 of MOXIe) [81]	No differences in primary outcome (peak work in exercise) but significant improvements in modified version of FARS (mFARS) were reported	NCT02255435 Phase II/III, randomized, placebo-controlled, double-blind, 48 weeks, parallel-group study is still ongoing (Part 2 of MOXIe). Announced results suggest improvement in mFARS [82,83]
Resveratrol	Anti-inflammatory Anti-apoptotic Neuroprotective Activates antioxidant defenses	1–5 g/day	Phase I/II Open-label clinical 12-week pilot study with two doses [84]	Frataxin levels remained unchanged. Lipid peroxidation markers decreased. FARS and ICARS scores improved. Gastrointestinal side effects reported	NCT03933163 Phase II, double-blind, placebo controlled 2-period crossover trial is currently ongoing with an enhanced formulation of resveratrol [85]
**Mitochondrial Metabolism**					
Pioglitazone	Increases PGC-1α Activates antioxidant defenses Reduces insulin resistance Improves energetic metabolism	15–45 mg/day	Phase III, prospective, randomized, double-blind, 2-years trial [86]	Pending publication	No further investigation
Leriglitazone (MIN-102)	Increases PGC-1α Restores mitochondrial function and bioenergetics Increases DRG neuron survival Improves motor function	Not specified	No previous clinical trials		NCT03917225 Phase II, randomized, double-blind, placebo-controlled study is currently ongoing (FRAMES) [87]
ALCAR	Mitochondrial cofactor Improves bioenergetic dysfunction Neuroprotective Anti-inflammatory Antioxidant	2–3 g/day	Placebo-controlled, 4-months, triple-phase crossover, creatine-ALCAR combined therapy [88]	No differences in Phosphocreatine, ICARS score or echocardiographic data compared to placebo	NCT01921868 Open-label trial of ALCAR with cardiovascular outcomes for 24 months. Results are pending publication [89]
**Iron Chelators**					
Deferiprone	Removes iron excess Enhances bioenergetic impairment Decreases ISCs damage	5–60 mg/kg/day	Several clinical trials have been conducted either with unique or combined therapy [90,91,92]	Improvements in cardiac outcomes, but no neurological effects were reported. Mild adverse effects such as neutropenia were observed	No further investigation
**Lipid Metabolism**					
EPI-743 (PTC-743)	15-lipoxigenase inhibitor CoQ_10_ improved antioxidant potential Anti-inflammatory Modulates energy metabolism	600 and 1200 mg/day	Phase II, Double-blind, with two phases and two doses. Placebo-controlled trial for 6 months and open-label for 18 months [93]	Safe and well tolerated. First phase: no differences in primary end point (visual acuity) but significant improvements in FARS score with high dose. Second phase: deceleration in severity of the disease, enhancing neurological function	Phase III, registrational trial is planned for 2020 [94]
RT001 (Retrotope)	Anti-inflammatory Reduces mitochondrial dysfunction, ROS production and ER stress	1.8–8.64 g/day	Phase I/II, double-blind placebo-controlled trial with two doses for 28 days [95]	Safe and well tolerated. Both doses improved cardiopulmonary and neurological tests.	NCT04102501 Phase III, randomized, double-blind, placebo-controlled trial is ongoing [96]

## Supplementary Materials

The following are available online at https://www.mdpi.com/2076-3921/9/8/664/s1, Table S1: Clinical trials with drugs whose main mechanism of action is not targeting oxidative stress.

## Abbreviations

ALCAR—l-Acetyl-carnitine	ARE—Antioxidant response element
dPUFAs—Deuterated PUFAs	DRG—Dorsal root ganglia
ER - Endoplasmic Reticulum	FARS—Friedreich Ataxia Rating Scale
FRDA—Friedreich’s ataxia	GPX4—Glutathione peroxidase 4
ICARS—International Cooperative Ataxia Rating Scale	ISC—Iron-sulphur clusters
KEAP1—Kelch-like ECH-associated protein 1	KIKO—Knock-in/knock out
mFARS—Modified Friedreich Ataxia Rating Scale	NRF2—Nuclear factor erythroid 2-related factor 2
NRFs—Nuclear respiratory factors	OXPHOS—Oxidative phosphorylation
PGC1-α—Peroxisome proliferator-activated receptor gamma coactivator 1-alpha	PPARγ—Peroxisome proliferator-activated receptor gamma
PPARs—Peroxisome proliferator-activated receptors	PUFAs—Polyunsaturated fatty acids
P-VEPs—Visual evoked potentials	ROS—Reactive oxygen species
SARA—Scale of Assessment and Rating of Ataxia	SIRT1—NAD-dependent deacetylase sirtuin-1
SOD2—Superoxide dismutase 2	SREBP1—Sterol-responsive element-binding protein 1
SWJs—Square-wave jerks	TZDs—Thiazolidinediones
